# The Practice of Medicine, a Treatise on Special Pathology and Therapeutics

**Published:** 1848-01

**Authors:** 


*The Practice of Medicine, a Treatise on Special Pathology and Therapeutics.* By Robley Dunglison, M. D., Professor of the Institutes of Medicine, &c., in Jefferson Medical College, Philadelphia; Lecturer on Clinical Medicine, &c. *Third Edition.* In two volumes, 8vo. pp. 1496. Lea & Blanchard, Philadelphia, 1848.

That a work like the present, on a subject of special science, in two large volumes, should pass into so many editions in a period of five years, is a rare compliment to the author. The third edition of a work so well appreciated, and from an author whose doctrines are so extensively known, requires but a brief notice. The author observes in the preface that, " Since the publication of the second edition of this work, short as the interval has been, so much activity has prevailed in the advancement of medical knowledge, that a thorough revision of it became necessary. Several pathological affections, too, had been omitted, which are now inserted." The volumes having come from the press since our journal was in the printer's hands, we have had but little time to glance through them; this superficial examination, however, has sufficed to show us that over two hundred pages have been added to the last edition, and every subject has shared in the revision. We think we may venture the assertion that, on every topic embraced in the work, the latest information will be found carefully posted up.

